# Prevalence and clustering of cardiovascular risk factors in a population aged 25–64 in Czechia: A cross-sectional study

**DOI:** 10.1371/journal.pone.0332217

**Published:** 2025-09-16

**Authors:** Juraj Michalec, Michala Lustigová, Anna Frűhaufová, Šárka Malá, Pavlína Krollová, Kristýna Žejglicová, Jana Malinovská, Lucia Bučková, Marika Koželuhová, Palavi Gupta, Pankhuri Jain, Natália Michalcová, Veronika Puchnerová, Matthew Campbell, Jan Brož

**Affiliations:** 1 Department of Internal Medicine, University Hospital Motol, Second Faculty of Medicine, Charles University, Prague, Czech Republic; 2 Department of Social Geography and Regional Development, Faculty of Science, Charles University, Prague, Czech Republic; 3 The National Institute of Public Health, Prague, Czech Republic; 4 Department of General Practice, Second Faculty of Medicine of Charles University, Prague, Czech Republic; 5 Department of Cardiology, University Hospital Motol, Second Faculty of Medicine, Charles University, Prague, Czech Republic; 6 Faculty of Health Science and Wellbeing, University of Sunderland, Sunderland, United Kingdom; National Healthcare Group, SINGAPORE

## Abstract

**Introduction:**

Cardiovascular events are still the most common cause of death in the Czech Republic. The increasing prevalence of risk factors such as dyslipidaemia, hypertension, obesity, and diabetes individually and collectively contribute to cardiovascular events. The aim of this study was to determine their prevalence and interrelationships.

**Method:**

The data for this epidemiological study were obtained from the Czech cross-sectional study EHES 2019 (European Health Examination Survey) with stratified random sampling. Firstly, individual risk factors (dyslipidaemia, hypertension, obesity, diabetes, and smoking) in population aged 25–64 years of age were monitored using questionnaires and physical and laboratory measurements; additionally, the cumulative effect of these risk factors in subjects was examined. Finally, cardiovascular risk in the age group of 40–64 years (767 out of 1057 participants) was estimated using the SCORE EU chart (for countries with high cardiovascular risk). Individual parameters were assessed according to standard criteria: dyslipidaemia = total cholesterol ≥5.0 mmol/l, and/or HDL-C < 1.0 mmol/l in men, or <1.2 mmol/l in women, and/or LDL-C ≥ 3.0 mmol/l and/or fasting TAG ≥ 1.7 mmol/l (or ≥2 mmol/l without fasting) and/or medication with lipid-lowering drugs; obesity = BMI > 30 kg/m^2^; hypertension = systolic blood pressure ≥ 140 mmHg, and/or diastolic blood pressure ≥ 90 mmHg, and/or antihypertensive treatment; diabetes mellitus = HbA1c ≥ 48 mmol/mol and/or on treatment. Data were analysed by descriptive statistics.

**Results:**

Of the total number of 1057 study participants (426 men and 631 women) aged 25–64 years, roughly 84% presented with ≥1 cardiovascular disease risk factor. The most common risk factor was dyslipidaemia, which occurred in 71.7% of the subjects. The prevalence of hypertension was 36.3% (men 46.0%, women 26.3%). 29.7% of subjects were obese, diabetes mellitus occurred in 5.7% (men 7.6%, women 3.7%). 17.7% were regular smokers; another 6.6% reported occasional smoking. A combination of risk factors was common, e.g., 77.3% had dyslipidaemia and/or hypertension. There were 16.1% of subjects without monitored cardiovascular risk factors. After stratification of cardiovascular risk prediction according to SCORE chart, 49.7% of individuals fall into low risk, 28.6% into medium risk, and up to 11.3% into high and 10.4% into very high risk.

**Conclusion:**

The most common risk factor is lipid spectrum disorders (71.7%). Combination of risk factors is common, which increases the risk of cardiovascular events in these individuals. In the 40–64 age group, 21.7% of the population is at >5% risk of a fatal cardiovascular event over the next 10 years.

## Introduction

Cardiovascular events are the leading cause of death worldwide [1,2], including the Czech Republic [3]. These include ischaemic heart disease, heart failure or brain stroke. Although cardiovascular mortality is slowly decreasing in the Czech Republic, cardiovascular morbidity is high and increasing, with cardiovascular diseases (CVD) affecting almost 3.5 million people [4]. A diagnosis of CVD is preceded by the prevalence of risk factors which are key targets for primary prevention.

Cardiovascular risk factors that have been well defined and studied include age, hypertension, obesity, dyslipidaemia, diabetes mellitus and smoking, most of which are preventable or manageable [5]. These risk factors are common although often undetected or untreated late into the course of CVD, with cardiovascular events sometimes being the first symptom. According to European Society of Cardiology the Czech Republic belongs to a high-risk region based on age- and sex-standardized CVD mortality rates [6]. The prevalence of CV risk factors in the Czech Republic was estimated in the study EHES (European Health Examination Survey) 2014 and 2019, in a working population aged 25–64 [7,8]. There has been a similar series of studies focusing on cardiovascular risk factors in the Czech Republic, the MONICA and post-MONICA studies.

SCORE is a risk-prediction model published in 2016 and widely used to estimate 10-year risk of fatal CVD in European populations. Our aim was to analyse the prevalence and relationships of CV risk factors in the Czech population based on the data obtained in EHES 2019 and stratify the studied population based on their estimated CVD risk according to SCORE models. Given the level of cardiometabolic health of the Czech population, the high-risk SCORE model was used [9,10].

## Methods

### Study subjects

Data from Czech EHES 2019 were used which captures a combination of self-assessed and clinically obtained data on non-communicable diseases and its associated risk factors among the Czech middle-aged population.

Study subjects were recruited among the study population of the European Health Interview Survey (EHIS). The methods of the EHIS are reported elsewhere [11], but in brief, the study comprises a cross-sectional survey conducted in a representative sample of 7995 participants older than 15 years living in the Czech Republic in 2019. It consisted of four modules: 1) health status (self-perceived health, chronic conditions, limitation in usual activities, disease-specific morbidity, physical and sensory functional limitations, etc.), 2) health care use (hospitalisation, consultations, unmet needs, use of medicines, preventive actions, etc.), 3) health determinants (height and weight, fruit and vegetable consumption, smoking, alcohol consumption, etc.) and 4) socio-economic background variables (sex, age, education, labour status, etc.). The data were collected via a professional interviewer-administered questionnaire. Of the 7995 participants, 4297 were aged 25–64 years of which 1057 participants completed the EHES 2019 health examination assessment [10,11].

### Measures

Information regarding sociodemographic characteristics (age, sex), lifestyle characteristics (e.g., smoking habits) were collected using health questionnaires, and anamnestic data (personal medical history of diabetes mellitus, hypertension, dyslipidaemia, current antidiabetic, antihypertensive, or lipid-lowering therapy) were collected using examination protocol. Blood pressure, blood cholesterol levels, level of HbA1c, TSH levels and presence of obesity were based on medical examination. The blood samples were not exclusively collected in a fasting state since non-fasting is now considered a standard approach in population-based screening studies.

During the health examination, the following measurements were made using standard procedures: weight, height, *waist circumference* (low risk: < 88 cm in women and < 102 cm in men), and systolic and diastolic blood pressure (SBP and DBP respectively). *Body mass index* (BMI) was calculated, participants with BMI of 25–29.9 kg/m^2^ were categorized as being overweight and those with BMI ≥ 30 kg/m^2^ were considered obese.

*Hypertension* was defined as systolic blood pressure (SBP) (mean of the second and the third measurements taken 1 min apart) ≥ 140 mmHg and/or diastolic blood pressure (DBP) measured repeatedly in a seated position (mean of the second and the third measurements taken 1 min apart) ≥ 90 mmHg and/or taking antihypertensive treatment [12].

For *dyslipidaemia* the risky level of total cholesterol (TC) was defined as ≥ 5 mmol/l and/or high-density lipoprotein (HDL) ≤ 1,2 mmol/l in men and ≤ 1 mmol/l in women and/or low-density lipoprotein (LDL) ≥3,0 mmol/l and/or triglycerides ≥1,7 mmol/l and/or lipid lowering therapy. All biochemical analyses were performed at the Czech Institute of Accreditation officially certified laboratories according to standardized procedures.

*Diabetes mellitus* was defined as HbA1c ≥ 48 mmol/mol and/or any treatment of previously diagnosed diabetes. Prediabetes (impaired fasting glucose and impaired glucose tolerance) was not included [13].

Current smoking was defined as smoking any tobacco products daily. Occasional smoking was defined as smoking any tobacco products less than daily, while not giving up smoking for good. Past smokers who have stopped smoking completely were not counted into this category.

### Cardiovascular risk stratification

Data from 767 participants aged 40–64 were analysed using SCORE models to estimate the cardiovascular risk, the probability of a fatal cardiovascular event in an individual during following 10 years. We used the models calibrated for high-risk countries, where the Czech Republic belongs to, based on age- and sex-standardized CVD mortality rate. Five variables are used in SCORE system: age, sex, current systolic blood pressure, cholesterol levels and smoking status. Although the model was primarily designed for clinical practice, it can also be used to quantify cardiovascular risk in the population and determine the distribution of risk levels. Individual risk values range from 0% to 47%. An important threshold is 5%; according to ESC recommendations, intervention should be initiated at or above this level. SCORE values were divided into four categories: low risk (0–1%), moderate risk (2–4%), high risk (5–9%), and very high risk (≥10%). Individuals who have already experienced cardiovascular disease or have diabetes are automatically considered to be at high risk, regardless of the SCORE chart value [9].

### Statistical analysis

Data are presented as prevalences of risk factors and as population means with standard deviations (SD) of the observed characteristics, weighted by age and sex. The distribution of categorical variables between sexes was assessed using the Chi-squared test, while differences in mean population values were evaluated using the independent samples t-test.

### Ethical considerations

Written informed consent was obtained from all EHES study participants before they underwent any study-specific procedures. The EHES study was conducted according to the applicable International Conference on Harmonisation (ICH)/Good Clinical Practice (GCP) standards and the World Medical Association Declaration of Helsinki – Ethical Principles for Medical Research Involving Human Participants and was approved by The National Institute of Public Health of the Czech Republic.

The data from the Czech EHES study were accessed on the 23^rd^ of June 2023 in a form that does not allow to identify individual participants during or after data collection.

## Results

From 4297 eligible participants enrolled in the EHIS study, a total of 1057 patients completed the laboratory tests in full (n = 426 male; 40.3%) of which 84% presented with at least one cardiovascular risk factor (see Table 1).

**Table 1:** Prevalence of cardiovascular risk factors among the Czech population aged 25–64 years, 2019, p-value for chi2-test.

CVD risk factors (prevalence in %)	Total	Males	Females	p-value
*Number of participants aged 25–64 years (N)*	*1057*	*426*	*631*	
Lipid disorder (dyslipidaemia)	71.7	73.8	69.6	0.130
Hypertension	36.3	46.0	26.3	<0.001
Obesity (BMI > 30 kg/m^2^)	29.7	32.9	26.3	0.018
Diabetes	5.7	7.6	3.7	0.005
Current smoking	17.7	18.3	17.2	0.617
Occasional smokers	6.6	8.4	4.7	0.015
Lipid disorder and/or hypertension	77.3	81.9	72.5	<0.001
Lipid disorder, hypertension and/or obesity	80.2	83.5	76.9	0.011
Lipid disorder, hypertension, obesity and/or diabetes	80.5	83.6	77.3	0.013
Lipid disorder, hypertension, obesity, diabetes and/or smoking	83.9	87.9	79.8	0.001
**Cardiovascular disease risk categories by SCORE EU chart (high risk), %**	**Total**	**Males**	**Females**	**p-value**
*Number of participants aged 40–64 years (N)*	*767*	*296*	*471*	
Very high, ≥ 10%	10.4	16.0	4.8	<0.001
High, 5–10%	11.3	19.2	3.2	
Moderate, 1–4%	28.6	35.9	21.1	
Low, < 1%	49.7	19.0	70.9	

Dyslipidaemia was the most common, present in 71.7% of participants and with an increased prevalence in men (73.8% vs. 69.6%); ~ 66% of cases were undiagnosed across both sexes. Only 26.0% of respondents with already known dyslipidaemia were on lipid-lowering treatment. Out of those treated, in only 23.7% total cholesterol, LDL, HDL and TAG was under target levels. Although there were almost twice as many men than women treated with hypolipidemics, the success rate of the therapy was reversed, with 32.1% of treated women meeting target values and only 19.1% of treated men with successful therapy.

The prevalence of hypertension was 36.3%. Hypertension prevalence was higher in men (46.0%) than women (26.3%). The awareness of hypertension was 60.1% in men and 72.0% women, with 42.0% men and 57.7% women of those being treated with antihypertensive agents. Similarly, treatment success rates were higher in women (60% vs 40%) meeting the target blood pressure ≤ 140/90 mmHg). The mean systolic blood pressure (SBP) was 131,4 (± 15,3) mmHg in men and 119,0 (± 15,9) mmHg. Only 17.9% of women and 4.6% of men achieved normal blood pressure (under 130/80 mmHg) on their therapy. The average values for both sexes are shown in Table 2.

**Table 2:** The average values of lipids and blood pressure for both sexes; p-value of the t-test.

Measured parameter	Total	Males	Females	p-value
Age, mean (SD)	44.5 (± 11.0)	44.3 (± 11.0)	44.7 (± 11.0)	0.485
Body mass index (kg/m^2^), mean (SD)	27.6 (± 5.4)	28.3 (± 4.7)	26.8 (± 6.0)	<0.001
Total cholesterol, mean (SD)	5.12 (±1.01)	5.08 (± 1.01)	5.16 (± 1.01)	0.204
LDL cholesterol, mean (SD)	3.03 (±0.92)	3.07 (± 0.92)	3.01 (± 0.93)	0.310
HDL cholesterol, mean (SD)	1.47 (±0.39)	1.33 (± 0.31)	1.63 (± 0.41)	<0.001
Non-HDL cholesterol, mean (SD)	3.65 (±1.02)	3.74 (± 1.01)	3.53 (± 1.02)	0.001
Triglycerides*, mean (SD)	1.45 (±1.11)	1.65 (± 1.30)	1.20 (± 0.74)	<0.001
SBP, mean (SD)	125.3 (± 16.8)	131.4 (± 15.3)	119.0 (±15.9)	<0.001
DBP, mean (SD)	81.5 (± 10.5)	83.8 (±10.4)	79.0 (±9.9)	<0.001

*Population means of triglycerides were computed only among fasting respondents.

Obesity was prevalent within the cohort with 29.7% of subjects classified as obese, with 32.9% of men and 26.3% of women. Additional 44.4% of men and 30.0% of women were overweight.

17.7% and 6.6% of respondents were active or occasional smokers, respectively; the prevalence was similar in both sexes. Diabetes (any type) was found in 5.7% of subjects with the prevalence twice as great in men as compared to women (7.6% of men, 3.7% of women).

A combination of risk factors was very common, e.g., 30.6% of respondents had dyslipidaemia and hypertension. There were only 16.1% of subjects without any of the studied cardiovascular risk factors.

Following SCORE stratification for the 10-year fatal cardiovascular event risk in those aged 40–64 years using charts for EU high risk country chart, the results were following: 49.7% of individuals fall into low risk category, 28.6% into medium risk, and up to 11.3% into high and 10.4% into very high risk category of a fatal cardiovascular event in the following 10 years.

## Discussion

From the results of the EHES 2019 study it is evident that cardiovascular risk factors are very common in the Czech Republic, even in a relatively young part of its population, with the most common being dyslipidaemia with overall prevalence of 71.7% (73.8% in men, 69.6% in women). This finding is consistent with the results of both EHES 2014 (77% in men, 66% in women) [14] and post-MONICA 2016/2017 (74.8% in men, 69.9% in women) [15,16]. Despite the high prevalence (consistent throughout the years) of dyslipidaemia, only 1/3 of the respondents had known about their condition, while only 26% of them were on lipid-lowering therapy and out of that only 23.7% reaching the target lipid levels. As such, of all the people with some form of dyslipidaemia (hypercholesterolemia being the most common), only 2% of them are treated adequately to reach target levels of blood lipids recommended for the general population.

Hypertension was found in 36.3% of participants, which is again comparable to EHES 2014 (34.6%) [14,17] and only slightly lower compared to post-MONICA 2016/2017 (41.5%) [14]. There are significant differences between sexes observed in all the studies, including our results, where the difference in mean SBP between sexes was 12.4 mmHg. An optimal blood pressure (under 120/80 mmHg) was found only in 16% of male and 43% of female participants. The awareness was, however, much better in contrast with dyslipidaemia and circa half of them were treated with antihypertensive drugs. Only 17.9% of women and 4.6% of men achieved normal blood pressure on their therapy. A possible explanation is that adherence to therapy could be lower in males. Nevertheless, as we have seen in the mean values of systolic blood pressure, there is approx. 10 mmHg difference in sexes. This trend is stable throughout the years [16], where even in the youngest age group of males (25–35 years) the mean BP has never been under 120/80 mmHg. Yet the definition for hypertension as well as the target values are universal for both sexes. Since the BP values are in proportion with cardiovascular risk, it is apparent that the males should be treated more aggressively to achieve the target BP.

Smoking habits were also studied in EHES 2019, with 17.7% regular smokers with not much of a difference between sexes (18.3% men and 17.2% women). An additional 7% reported occasional smoking. Here we can see a decline in the number of smokers in the past 5 years, since in EHES 2014 there was 25.7% of regular smokers (27% of men, 24.3% of women) and additional 6% of occasional smokers. This is consistent with an observed trend of decreasing number of people who smoke in the Czech Republic, with comparable results in post-MONICA studies and NAUTA studies concluded regularly by National Institute of Public Health (SZÚ) [18]. A steeper decline is observed in male population, with women somewhat stagnant or even rising, especially a few decades ago [16]. As tobacco smoke is considered not only a significant cardiovascular risk factor, but an important carcinogen as well, this trend is also reflected in the incidence of lung cancer in the Czech Republic – a decreasing incidence in men and increasing incidence in women with a stabilising trend in the past few years [19]. While the general trends in smoking seem favourable, there is indeed a new concern about the rising abuse of alternative tobacco products, such as e-cigarettes or vape products, especially among teenagers and adolescents – a population mostly not reflected in EHES study. The numbers are quite alarming worldwide, with 30–45% of teenagers ever-using e-cigarettes [20], as well as in the Czech Republic, where the latest reports show that in adolescents aged 15–24 about half of them use nicotine in some form while 30% of them use it daily [21].

The major limit of this study is a volunteer bias, where, as mentioned above, only about one fourth of plausible subjects voluntarily participated in all the necessary tests of EHES study, which could lead to skewed results. When it comes to smoking prevalence, reporting bias can contribute to underestimation.

## Conclusion

The results of this study show that despite focusing on younger, productive population, the prevalence of the most important cardiovascular risk factors remains unacceptably high. Our findings show that the most common and most underestimated risk factor is dyslipidemia, where the awareness of patients as well as target levels attainment is unsatisfactory. We should therefore focus more on primary prevention in both screening and treatment of dyslipidemia.

The results of this study are highly congruent with previous epidemiological studies performed in the Czech Republic or Central Europe in general and we see a slightly declining prevalence in some risk factors, mainly hypertension [16]. The prevalence of diabetes was a little lower in this study compared to previous studies [14,17], this observed variability can be attributed to low prevalence of diabetes mellitus in the studied population, thus is largely dependent on the selected sample of that population. The variability of high-prevalence factors is significantly lower.

In conclusion, the results of EHES 2019 prove that even younger populations could be at a significant cardiovascular risk and should encourage doctors across specialities to screen for and address the present risk factors accordingly. Doing that we can continue in the declining trend in some of the risk factors and, most importantly, prevent major cardiovascular events at young age.

## References

[1] Global health estimates: Leading causes of death [Internet]. WHO; 2025 [cited 2025 Mar 22]. Available from: https://www.who.int/data/gho/data/themes/mortality-and-global-health-estimates/ghe-leading-causes-of-death

[2] Ahmad FB, Anderson RN. The Leading Causes of Death in the US for 2020. JAMA. 2021;325(18):1829–30. doi: 10.1001/jama.2021.5469 33787821 PMC8145781

[3] Zemřelí podle zkráceného seznamu příčin smrti v ČR a krajích, pololetní data - 2019–2023 [Internet]. Český statistický úřad; 2024 [cited 2025 Mar 22]. Available from: https://csu.gov.cz/produkty/zemreli-podle-zkraceneho-seznamu-pricin-smrti-v-cr-a-krajich-pololetni-data-ozhketasyv

[4] Ústav zdravotnických informací a statistiky ČR. Zátěž populace ČR kardiovaskulárními onemocněními [Internet]. Národní zdravotnický informační portál; 2024 [cited 2025 Mar 22]. Available from: https://www.nzip.cz/data/1666-kardiovaskularni-onemocneni-zatez-ceska-republika

[5] Ciumărnean L, Milaciu MV, Negrean V, Orășan OH, Vesa SC, Sălăgean O, et al. Cardiovascular Risk Factors and Physical Activity for the Prevention of Cardiovascular Diseases in the Elderly. Int J Environ Res Public Health. 2021;19(1):207. doi: 10.3390/ijerph19010207 35010467 PMC8751147

[6] European Risk Regions [Internet]. HeartScore; 2021 [cited 2025 Mar 22]. Available from: https://www.heartscore.org/en_GB/heartscore-europe-risk-regions

[7] European Health Examination Survey [Internet]. EHES; 2009-2013 [cited 2025 Mar 22]. Available from: https://www.ehes.info/

[8] Čapková N, Lustigová M. Zdravotní stav české populace - výsledky studie EHES 2019 [Internet]. SZÚ; 2023 [cited 2025 Mar 22]. Available from: https://szu.gov.cz/temata-zdravi-a-bezpecnosti/studie-zdravotniho-stavu-obyvatelstva/dospeli/ehes/ehes-2019/

[9] Piepoli MF, Hoes AW, Agewall S, Albus C, Brotons C, Catapano AL, et al. European Guidelines on cardiovascular disease prevention in clinical practice. Europe J Prevent Cardiol [Internet]. 2016 [cited 2025 Mar 22];23(11):NP1–96. Available from: https://academic.oup.com/eurjpc/article/23/11/NP1-NP96/5927332

[10] About HeartScore [Internet]. HeartScore; 2021 [cited 2025 Mar 22]. Available from: https://www.heartscore.org/en_GB/about-heartscore

[11] European health interview survey [Internet]. Eurostat; 2024 [cited 2025 Mar 22]. Available from: https://ec.europa.eu/eurostat/web/microdata/european-health-interview-survey

[12] Hypertension [Internet]. WHO; 2023 [cited 2025 Mar 22]. Available from: https://www.who.int/news-room/fact-sheets/detail/hypertension

[13] American Diabetes Association. 2. Classification and Diagnosis of Diabetes: Standards of Medical Care in Diabetes—2018. Diabetes Care [Internet]. 2018 [cited 2025 Mar 22];41(Supplement_1):S13–S27. Available from: https://diabetesjournals.org/care/article/41/Supplement_1//30088/2-Classification-and-Diagnosis-of-Diabetes 10.2337/dc18-S002 29222373

[14] Zdravotní stav české populace - výsledky studie EHES 2014 [Internet]. SZÚ; 2018 [cited 2025 Mar 22]. Available from: https://szu.gov.cz/temata-zdravi-a-bezpecnosti/studie-zdravotniho-stavu-obyvatelstva/dospeli/ehes/ehes-2014/

[15] Cífková R, Bruthans J, Wohlfahrt P, Krajčoviechová A, Šulc P, Eremiášová L, et al. The prevalence of major cardiovascular risk factors in the Czech population in 2015-2018. The Czech post-MONICA study. Cor et Vasa [Internet]. 2020 [cited 2025 Mar 22];62(1):6–16. Available from: http://e-coretvasa.cz/doi/10.33678/cor.2020.010.html

[16] Cífková R, Bruthans J, Wohlfahrt P, Krajčoviechová A, Šulc P, Jozífová M, et al. 30-year trends in major cardiovascular risk factors in the Czech population, Czech MONICA and Czech post-MONICA, 1985 - 2016/17. PLoS One. 2020;15(5):e0232845. doi: 10.1371/journal.pone.0232845 32392239 PMC7213700

[17] Vejtasová V, Lustigová M, Urbanová J, Žejglicová K, Malinovská J, Janíčková Žďárská D, et al. Prevalence and management of arterial hypertension in the population aged 25-64 in the Czech Republic with a focus on diabetic patients. Epidemiol Mikrobiol Imunol. 2021;70(4):247–52. 35073703

[18] NAUTA – Národní výzkum užívání tabáku a alkoholu v České republice [Internet]. SZÚ; 2023 [cited 2025 Mar 22]. Available from: https://szu.gov.cz/odborna-centra-a-pracoviste/centrum-podpory-verejneho-zdravi/prevence-zavislosti/studie/nauta/

[19] Borilova S, Dusek L, Jakubikova L, Turcani P, Matej R, Hanke I, et al. Lung Cancer in the Czech Republic. J Thorac Oncol. 2023;18(3):271–7. doi: 10.1016/j.jtho.2022.11.023 36842812

[20] Lyzwinski LN, Naslund JA, Miller CJ, Eisenberg MJ. Global youth vaping and respiratory health: epidemiology, interventions, and policies. NPJ Prim Care Respir Med. 2022;32(1):14. doi: 10.1038/s41533-022-00277-9 35410990 PMC9001701

[21] NAUTA 2023: Přibývá mladých kuřáků elektronických cigaret, polovina Čechů mezi 15-24 lety užívá nikotin [Internet]. SZÚ; 2024 [cited 2025 Mar 22]. Available from: https://szu.gov.cz/tiskove-zpravy/nauta-2023-pribyva-mladych-kuraku-elektronickych-cigaret-polovina-cechu-mezi-15-24-lety-uziva-nikotin/

